# Population-Level Heterogeneity in People with Obesity: A Cross-Sectional Cluster Analysis of a Population-Based Registry

**DOI:** 10.3390/jcm15187026

**Published:** 2026-09-10

**Authors:** Orly Tamir, Dor Hadida Barzilai, Havi Murad, Nirit Agay, Leah Borovoi, Avraham Karasik, Ariel Israel, Eugene Merzon

**Affiliations:** 1 Pesach Segal Israeli Center for Diabetes Research and Policy, Sheba Medical Center, Sheba Rd. 2, Ramat Gan 5262100, Israel; dorh1994@gmail.com (D.H.B.); israelev@gmail.com (L.B.); 2 National Diabetes Council, Ministry of Health, Jerusalem 9101002, Israel; 3 Data & Analytics Division, Sheba Medical Center, Sheba Rd. 2, Ramat Gan 5262100, Israel; havim@gertner.health.gov.il (H.M.); nirita@gertner.health.gov.il (N.A.); 4 Gray Faculty of Medical and Health Sciences, Tel Aviv University, Haim Levanon St. 55, Ramat Aviv, Tel Aviv 6997801, Israel; karasik@tauex.tau.ac.il (A.K.); aisrael@leumit.co.il (A.I.); 5 Leumit Health Services, Shprintzak 23, Tel Aviv 6473817, Israel; emerzon@gmail.com; 6 The Adelson School of Medicine, Ariel University, Kiryat HaMada St. 3, Ariel 4070000, Israel

**Keywords:** obesity, population-based registry, cluster analysis, obesity-treatment utilization

## Abstract

**Background**: Obesity is a heterogeneous chronic disease, yet it is still commonly defined and managed using body mass index alone. Identifying clinically meaningful subgroups may support more efficient, precise and practical care. **Objective**: To identify and characterize population-level heterogeneity among adults with obesity in a large Israeli healthcare cohort. **Methods**: In this cross-sectional study, we analyzed deidentified electronic health record data from the Leumit Obesity Registry. Adults with obesity who had at least one documented weight measurement and height or BMI record during 2024 were included. Demographic, socioeconomic, lifestyle, clinical, and treatment variables were analyzed using K-prototypes cluster analysis to identify subgroups within a mixed-data population. **Results**: The study included 68,203 adults with obesity. Five distinct patient clusters were identified: metabolically healthy young adults, 19% of the cohort; adults with low comorbidity, 18%; middle-aged adults with moderate comorbidity, 23%; multimorbid seniors with socioeconomic disadvantage, 24%; and higher socioeconomic status advanced age with high clinical burden, 16%. The clusters differed substantially in age, comorbidity burden, socioeconomic status, and obesity-treatment utilization. GLP-1 use was most common in the older multimorbid clusters, particularly among low SES. Dietitian use was lower in the oldest and sickest cluster, despite high disease burden. Bariatric surgery was overall relatively rare and was concentrated mainly in the younger clusters. **Conclusions**: Adults with obesity in this large population-based registry did not represent a single clinical group, but rather several distinct phenotypes with different clinical and treatment patterns. These findings support a shift toward more phenotype-informed obesity care and resource planning.

## 1. Introduction

Obesity is a global pandemic affecting approximately 16% of the adult population worldwide and is associated with severe multimorbidity [1]. Yet body mass index (BMI) alone does not adequately capture the clinical and metabolic heterogeneity of obesity and is insufficient as a stand-alone guide for individual diagnosis and treatment stratification [2,3,4,5]. Obesity is increasingly conceptualized as a heterogeneous adiposity-based chronic disease, encompassing diverse clinical phenotypes and complication burdens, which supports a move away from a “one-size-fits-all” therapeutic approach [3,6,7,8].

To address this complexity, recent research has increasingly utilized unsupervised machine learning techniques to move beyond traditional classifications. Studies in diverse healthcare settings, including the UK, Japan, and France, have successfully applied cluster analysis to partition obesity into distinct clinical phenotypes, demonstrating that patient subgroups differ significantly in their risk profiles, health burden, lifestyle patterns, and response to medical and surgical interventions [5,9,10,11]. However, these phenotypes are highly sensitive to local demographic, cultural, and socioeconomic contexts, meaning models derived from foreign populations cannot be automatically extrapolated to other populations. Furthermore, there is a lack of large-scale data examining how such theoretical clusters align with real-world treatment utilization, such as the prescription of GLP-1 analogs or access to bariatric surgery, in a community setting.

In the present study, our objective was to identify and characterize obesity-related population-level heterogeneity within the Israeli healthcare system. Specifically, we sought to determine whether distinct phenotypes based on demographic, clinical, behavioral, and socioeconomic characteristics could be identified among adults with obesity. Identifying such phenotypes may have clinical relevance by highlighting patient groups with different combinations of disease burden, metabolic and behavioral characteristics, socioeconomic vulnerability, and healthcare utilization that may not be adequately distinguished by BMI alone. Characterizing these groups may support more clinically informed risk stratification and treatment planning, while also helping health systems identify differences in treatment utilization and unmet needs, inform targeted resource allocation, and generate hypotheses for more individualized obesity care.

## 2. Methods

### 2.1. Study Design, Setting, and Population

This cross-sectional study was conducted from May to June 2025. Reporting was guided by the Strengthening the Reporting of Observational Studies in Epidemiology (STROBE) recommendations for cross-sectional studies, and the completed checklist is provided in Supplementary Table S1.

The study population was drawn from the Leumit Obesity Registry of the Leumit Healthcare Services (LHS). LHS is the fourth largest healthcare provider in Israel insuring 778,032 individuals as of 2025 (7.35% of the Israeli population) [12]. LHS has a comprehensive computerized database incorporating demographics, medical diagnoses, primary and secondary care medical visits, hospitalizations, and laboratory tests. All LHS members hold similar health insurance and similar access to healthcare services. During each physician visit, a diagnosis is entered or updated according to the International Classification of Diseases 9th revision (ICD-9). The Leumit Obesity Registry [13] is the first population-based comprehensive obesity registry in Israel, which was established in 2022 for identification and target management of high-risk patients. The development of the registry was based on a retrospective review of electronic health records data from all LHS enrollees, identifying adult individuals with obesity, defined as a BMI ≥ 30 kg/m^2^. Eligible participants in the study were adults included in the Leumit Obesity Registry, who had at least one BMI measurement or weight measurement recorded during the 2024 study year. Registry members who did not have a qualifying measurement during 2024 were not included in the analytic cohort. No additional sampling was performed. In February 2025, the registry included 114,028 individuals. Among them, 68,203 had a documentation of at least one BMI value or weight measurement during the year 2024, which was defined as the study year being the most recent complete calendar year for which the registry contained sufficiently mature and consistently recorded data. All eligible individuals meeting the pre-specified registry-based criteria were included; therefore, no sample-size calculation or sampling procedure was performed. The study was approved by the IRB of the Sheba Medical Center (approval number SMC-0730-23, dated 8 November 2023) and Leumit Healthcare Services (approval number LEU-0031-23, dated 3 November 2024).

### 2.2. Variables

This study was performed using de-identified electronic health records. Data for analysis were all drawn from the Obesity Registry and included demographic information, lifestyle, anthropometric and laboratory measurements, comorbidities, and obesity treatments. Demographic variables were age (continuous variable), sex (binary variable), ethnicity (general Jewish, Orthodox/ultra-orthodox Jewish, Arab), and socioeconomic status (4 commonly used categories based on residence address SES scores of the Israel Central Bureau of Statistics). Lifestyle variables included self-reported status of smoking (binary variable) and amount of weekly physical activity (categories: no physical activity, occasional, 1–3 times per week, more than 3 times per week). Comorbidities included all documented conditions in the medical record (binary variable). Finally, we included a binary factor to each of the following obesity treatments that were utilized during 2024: consultation with a dietician, participation in group weight loss seminar, weight loss medication, and bariatric surgery.

### 2.3. Analysis

We applied K-prototypes cluster analysis to identify distinct subgroups of individuals with obesity in our dataset. The analysis included categorical variables: sex, ethnic sector, smoking status, physical activity, and socioeconomic status (SES), and numerical variables: age, Charlson Index score, and BMI. The selection of clustering variables was guided primarily by the objective of identifying population-level, clinically and socially meaningful profiles of people with obesity using routinely available registry information. Variables with missing data (e.g., SES) were treated as categorical by including a separate “missing” category.

The K-prototypes algorithm extends the traditional K-means approach by incorporating elements of the K-modes algorithm, enabling clustering on mixed-type data. It minimizes a combined cost function that uses squared Euclidean distance for numerical variables and simple matching dissimilarity for categorical variables [14].

Prior to clustering, we standardized all numerical variables and initialized cluster centroids using Huang’s method [14]. The optimal number of clusters was determined using the elbow method applied to the cost curve for cluster numbers ranging from 3 to 10, supplemented by the additional metrics R-squared and Cubic Clustering Criterion (CCC). To quantify the magnitude of between-cluster differences for each input variable, ANOVA effect sizes were calculated for all variables included in the clustering analysis [15]. For continuous variables, omega squared (ω^2^) was used to quantify the magnitude, whereas Cramér’s V was used for categorical variables.

Later, descriptive statistical analyses were performed for each cluster.

Analyses were conducted using the kmodes package in Python 3.12 [16].

## 3. Results

### 3.1. Study Population

The study population included a total of 68,203 adult patients identified in the Leumit Health Services obesity registry as of February 2025. Inclusion was based on having at least one weight measurement and a documentation of height, or BMI documentation during the year 2024. Mean age of the study population was 52.45 (SD 17.59), mean BMI 34.61 (SD 4.26), 60.2% Females, 54.4% General Jewish, 19.9% Jewish Ultra-Orthodox, and 25.7% Arabs.

### 3.2. Identification of Obesity Clusters

**Figure 1:**
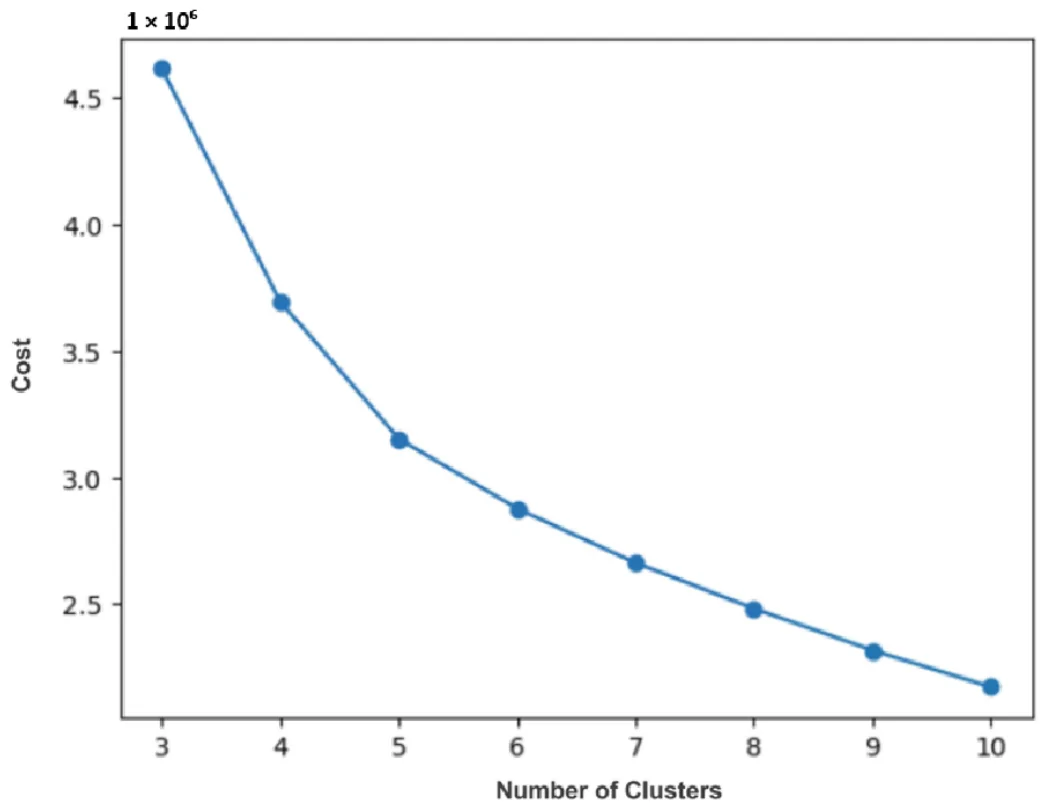
Elbow Plot for Determining the Optimal Number of Clusters in K-Prototypes Clustering.

Using K-prototypes clustering analysis, based on the joint distribution of demographic, lifestyle, and clinical characteristics, five distinct patient profiles were identified. Figure 1 presents the total cost (sum of within-cluster dissimilarities) for k-prototypes clustering solutions ranging from 3 to 10 clusters. The number of clusters was primarily determined based on the clustering cost from the k-prototypes analysis, which accounts for both numeric and categorical variables. A sharp decline in cost was observed from three to five clusters (4,615,180 to 3,152,489), after which the rate of decrease flattened, indicating diminishing returns with the addition of further clusters. The five-cluster solution was further supported by the clustering statistics calculated for the numeric variables. R^2^ increased from 0.794 for three clusters to 0.862 for five clusters, with progressively smaller improvements for subsequent cluster solutions (0.875–0.907 for six to ten clusters). Similarly, the Cubic Clustering Criterion (CCC) was −1.035 for five clusters and became progressively more negative with additional clusters. Taken together, the cost criterion, which incorporates both numeric and categorical variables, and the supplementary numeric-variable statistics, supported the selection of five clusters as a parsimonious solution for subsequent analyses. Full data on cluster validation indices are provided in Supplementary Table S2 and Figure S1. The distribution of patients across these clusters ranged from 16% to 24% of the total study population, indicating relatively balanced cluster sizes.

### 3.3. Characteristics of Patient Profiles

The five identified clusters exhibit distinct demographic and clinical characteristics (Table 1 and Table 2). The cluster names are descriptive labels summarizing the predominant characteristics of each cluster and are intended to facilitate interpretation. They should not be interpreted as implying that all individuals within a cluster share all of the characteristics.

**Cluster 1: Metabolically Healthy Young Adults**

This cluster comprised 19% (n = 12,997) of the population. It represents the youngest cohort, with a mean age of 26.38 ± 4.38 years. Mostly female (65%), non-smokers (82%) and low SES (46%). Patients in this group had the lowest comorbidity burden (mean Charlson Comorbidity Index = 0.07 ± 0.03) and very low rates of chronic conditions such as hypertension (3%) and diabetes (3%).

**Cluster 2: Adults with Low Comorbidity**

Comprising 18% (n = 12,159) of the cohort, this group had a mean age of 40 ± 3.75 years. While still relatively healthy (mean Charlson Index = 0.72 ± 0.86), this group showed a slight increase in chronic conditions compared to Cluster 1, with 12% prevalence of hypertension and 13% prevalence of diabetes.

**Cluster 3: Middle-Aged with Moderate Comorbidity**

This cluster included 23% (n = 15,793) of the population. The mean age was 53.15 ± 3.65 years. This group exhibited a moderate disease burden (mean Charlson Index = 2.44 ± 1.23), with significantly higher rates of hypertension (38%) and diabetes (31%) compared to the younger clusters.

**Cluster 4: Multimorbid Senior Adults with Socioeconomic Disadvantage**

This was the largest cluster, representing 24% (n = 16,669) of the population. The mean age was 65.52 ± 3.36 years. This group is characterized by high comorbidity (mean Charlson Index = 4.29 ± 1.52). Notably, this group had a high proportion of patients with low SES (44%).

**Cluster 5: High Clinical Burden Advanced-Age with higher SES**

Representing 16% (n = 10,585) of the cohort, this cluster included the oldest and sickest patients (Mean age 77.2 ± 4.95 years; mean Charlson Index = 6.49 ± 2.09). This group was predominantly from the General Jewish sector (78%) and had the lowest rate of current smokers (8%), higher SES, and highest rates of hypertension (85%), dyslipidemia (81%), COPD (16%), CVA (8%), and DM with end organ damage (22%).

### 3.4. Magnitude of Differentiation Between the Clusters

The calculation of effect sizes for all variables included in the clustering analysis revealed that age demonstrated the largest between-cluster effect (ω^2^ = 0.75), with mean age ranging from 26.4 years in Cluster 1 to 77.2 years in Cluster 5. The Charlson Comorbidity Index also showed a very large between-cluster effect (ω^2^ = 0.56), with mean scores ranging from 0.07 to 6.49 across clusters. Among categorical variables, ethnic sector demonstrated a moderate association with cluster membership (Cramér’s V = 0.22), while socioeconomic status showed a smaller but meaningful association (V = 0.14). Smaller associations were observed for smoking status (V = 0.10), physical activity (V = 0.06), and sex (V = 0.06). BMI demonstrated minimal between-cluster differentiation (ω^2^ = 0.01). These findings indicate that although age was the strongest differentiating characteristic of the cluster solution, the clusters also differed substantially in comorbidity burden and, to a lesser extent, demographic (ethnic sector), socioeconomic, and behavioral characteristic (smoking).

**Figure 2:**
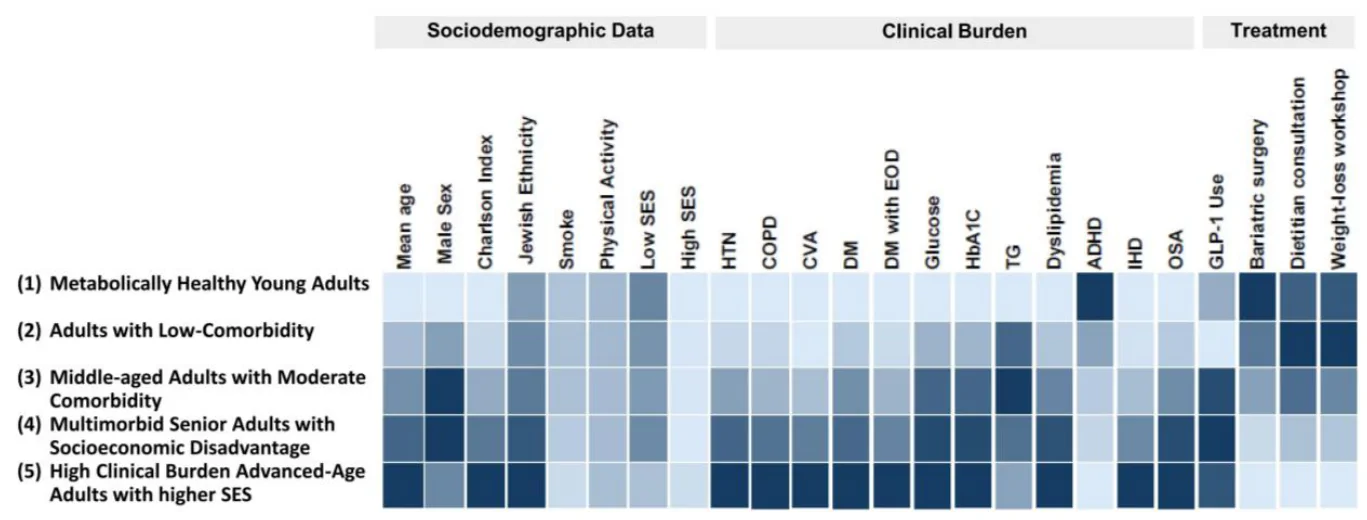
Magnitude of multidomain characteristics across obesity clusters. Color intensity represents the relative magnitude of each characteristic across clusters, with lighter blue indicating lower values and darker blue indicating higher values.

A heat map in Figure 2 provides a visual representation of the magnitude of characteristics across patient clusters.

### 3.5. Treatment Utilization Patterns

Analysis of treatment utilization in 2024 revealed distinct patterns across clusters (Table 3):

**Pharmacotherapy (GLP-1):** GLP-1 analogs were the most common intervention, utilized by 10,888 individuals of 68,203 patients (16.0%). The highest utilization was observed among multimorbid low SES senior adults (Cluster 4), comprising 3551 individuals (21.2% of the cluster population), followed by middle aged individuals with moderate comorbidity (Cluster 3), with 3140 individuals (19.9%). In contrast, utilization was lower in the younger, lower-comorbidity clusters, with 11.2% (1460/12,997) in the “Metabolically Healthy Young Adults” group and 5.9% (716/12,159) in “Adults with Low Comorbidity”. Despite similar cluster sizes, GLP-1 utilization in the “Metabolically Healthy Young Adults” was nearly double that in the “Adults with Low Comorbidity”.

**Dietary counseling:** Nutritional counseling was documented for 11,089 individuals out of 68,203 (16.3%) comprising the study population. Utilization was relatively evenly distributed among Clusters 1 to 3, ranging from 18.0% to 20.9%. In contrast, lower rates were observed in the older and more medically complex clusters, with 12.9% (2157/16,669) in Cluster 4 and 10.5% (1109/10,585) in Cluster 5.

**Group weight-loss seminar:** Participation in group weight-loss seminars was low overall, at 3.4% of the study population (2300), and declined with increasing age and comorbidity. Rates were 4.5% (589/12,997) in Cluster 1, 5.1% (622/12,159) in Cluster 2, 3.6% (563/15,793) in Cluster 3, 2.2% (372/16,669) in Cluster 4, and 1.5% (154/10,585) in Cluster 5.

**Bariatric surgery:** Surgical intervention was rare overall, with only 340 cases recorded in 2024, representing 0.5% of the cohort. The majority of surgeries were performed in the youngest cluster (n = 148, representing 1.1% of individuals in this cluster), with rates declining with age and comorbidity.

## 4. Discussion

This study utilized a machine-learning clustering approach to identify five distinct phenotypes of obesity within a large Israeli cohort of 68,203 patients. The analysis confirmed that obesity is a highly heterogeneous condition, with patient profiles varying significantly by age, comorbidity burden, and socioeconomic status (SES). Although age was the strongest differentiating characteristic across clusters, the clustering solution we suggest also captured meaningful variation in comorbidity burden, ethnic sector, socioeconomic status, and other and behavioral characteristics, indicating that the identified phenotypes were not defined by age alone. This interpretation is consistent with contemporary obesity frameworks that emphasize heterogeneity, complication-oriented care, and the need to translate obesity science into individualized clinical practice rather than relying on uniform management strategies [3,6].

Unlike BMI, which primarily reflects body size, or the Edmonton Obesity Staging System (EOSS), which stages disease severity based on obesity-related complications [17], unsupervised clustering integrates multiple clinical dimensions simultaneously and identifies patient subgroups with shared metabolic, behavioral, healthcare utilization, and socioeconomic characteristics [15]. This multidimensional approach may reveal clinically meaningful heterogeneity among individuals with similar BMI values or comparable EOSS stages, potentially enabling more refined risk stratification and tailored management strategies. Rather than replacing established classification systems, clustering may serve as a complementary framework that supports precision medicine by identifying patients who may benefit from different monitoring strategies, multidisciplinary interventions, or therapeutic priorities.

### 4.1. The “Metabolically Healthy Young Adults” Phenotype and Early Intervention

We identified a distinct cluster of “Metabolically Healthy Young adults” (Cluster 1), representing 19% of the study population (n = 12,997). These patients were characterized by a very young age, mostly females (65%), and a near absence of comorbidities. This finding mirrors the “Younger healthy females” cluster described by Green et al. [10], which comprised the largest subgroup in their UK cohort, and “Cluster 3” (healthy young women) identified by Borel et al. [9] in a bariatric cohort.

The identification of this metabolically healthy phenotype presents a critical window for intervention before substantial cardio-metabolic disease accumulates [9,10,11]. Borel et al. demonstrated that this specific profile (young, healthy women) achieved the best total body weight loss outcomes following bariatric surgery [9]. However, our data indicates that bariatric surgery utilization in this cluster is low (n = 148, 1.1%) relative to the population size, though it is higher than in the older, more complex clusters. The challenge for this group is preventing the progression to metabolic disease. As noted by Green et al. [10], interventions for this demographic might be more effective when focused on lifestyle modification, prevention, and long-term risk reduction rather than intensive disease management required for patients with established complications.

### 4.2. Intermediate Phenotypes and the Continuum of Clinical Burden

Clusters 2 and 3 illustrate intermediate levels of clinical burden between the lowest-burden and highly multimorbid groups. These findings show that adults with the same BMI-defined obesity may have substantially different combinations of comorbidities, socioeconomic characteristics, behaviors, and healthcare utilization. They should not, however, be interpreted as evidence of sequential progression between clusters.

The act of clustering is complementary to, rather than a replacement of, established approaches such as BMI and EOSS. Whereas BMI reflects body size and EOSS stages obesity-related health impairment, clustering captures recurring multidimensional profiles that may help identify groups with different care needs at the population and health-system level.

### 4.3. The Burden of the “Sick and Disadvantaged” Phenotype

Perhaps the most clinically significant finding is the identification of Cluster 4 (“Multimorbid Seniors with Socioeconomic Disadvantage”), which constitutes the largest segment of our cohort (24%). This group combined a high burden of diabetes and hypertension with marked socioeconomic disadvantage. This profile strongly resembles the “Poorest health” cluster identified by Green et al. [10], and it aligns with prior work showing that obesity subgroups with greater clinical complexity tend to experience worse overall health status and, in surgical cohorts, less favorable outcomes [9,10]. The relationship between socioeconomic status, age, and clinical burden warrants particular consideration. In cluster 4, the coexistence of older age and socioeconomic deprivation may contribute to the greater accumulation of multimorbidity observed in this group. Socioeconomic disadvantage may compound the effects of aging through prolonged exposure to barriers related to access to healthcare, health-promoting resources, disease prevention, and effective long-term disease management. Although the cross-sectional nature of our study does not permit causal inference, this phenotype may reflect the cumulative interplay between socioeconomic disadvantage and advancing age over the life course. Social disadvantage is a well-established driver of health inequalities [18]. One plausible mechanism is lower health literacy, which has been linked to socioeconomic disadvantage and may reduce engagement with preventive and chronic disease care [19,20]. Taken together, these findings suggest that this phenotype may require not only intensified medical treatment but also socially responsive models of care that incorporate multidisciplinary support and address barriers to sustained obesity management beyond clinical treatment alone.

In our cohort, this high-risk group (Cluster 4) had the highest utilization of GLP-1 analogs. This observation likely reflects prioritization of pharmacotherapy in patients with greater metabolic burden, but it should not be interpreted as evidence of greater treatment responsiveness. Current guidance frames GLP-1 therapy as one component of long-term, integrated, person-centered obesity care, and recent pharmacologic frameworks emphasize aligning treatment choice with the patient’s complication profile rather than viewing medication use in isolation [21,22].

### 4.4. Treatment Utilization Gaps

Treatment utilization varied substantially across the identified phenotypes. Nutritional counseling was relatively common in the middle-aged (intermediate) clusters (Clusters 3 and 2) but notably lower in the oldest and sickest phenotype, despite the very high comorbidity burden in that group (Cluster 5). This pattern may reflect implementation gaps in delivering obesity care to medically complex patients, in whom competing illnesses, treatment burden, and reduced care capacity can limit uptake of guideline-concordant interventions [6,23]. Accordingly, the lower use of dietary counseling in Cluster 5 may represent a missed opportunity to support the management of comorbidities such as hypertension, which affected 85% of this cluster. However, this interpretation is limited by the lack of information on the indication for individual dietitian encounters. In patients with high multimorbidity, consultations may have been related to diabetes, renal disease, cardiovascular risk, or other conditions rather than obesity specifically.

GLP-1 receptor agonist exposure was highest in Cluster 4. However, the indication for GLP-1 treatment could not be determined from the available data. Because diabetes was substantially more prevalent in the older, multimorbid clusters, the higher GLP-1 exposure in these groups may partly or largely reflect treatment of diabetes rather than obesity-specific pharmacotherapy. These findings should therefore be interpreted as patterns of GLP-1 exposure, irrespective of indication, rather than as differences in obesity-treatment uptake.

Bariatric surgery was uncommon overall and was concentrated mainly in the younger clusters. This contrasts with the bariatric cohort reported by Borel et al., where all patients underwent surgery and outcomes varied by phenotype [9]. In our data, the low surgical volume in the “Adults with Low Comorbidity” cluster may suggest underuse of an effective modality in a subgroup that is still early enough in its disease course to potentially derive substantial long-term benefit, although this interpretation should remain cautious because our study did not assess surgical eligibility, contraindications, or patient preference.

Since cluster membership was derived from demographic, lifestyle, socioeconomic, anthropometric, and comorbidity variables, multivariable adjustment for these same characteristics was not performed, as this would have adjusted for variables used to define the exposure of interest. Accordingly, treatment comparisons should be interpreted as descriptive and hypothesis-generating.

### 4.5. Positioning Our Results Within ABCD and EOSS Frameworks

Our findings can be further contextualized within the Adiposity-Based Chronic Disease (ABCD) model and the Edmonton Obesity Staging System (EOSS), both of which move beyond BMI-centric definitions toward a complications-oriented understanding of obesity. The ABCD framework conceptualizes obesity as a chronic, progressive disease driven by excess or dysfunctional adiposity and defined primarily by the presence, severity, and trajectory of complications rather than body size alone [3,24]. Similarly, EOSS stratifies patients into stages (0–4) based on metabolic, physical, and psychological complications, providing a clinically actionable method for risk stratification and treatment prioritization (217).

In this context, the phenotypic clusters identified in our study can be interpreted as data-driven analogues of EOSS stages. For example, Cluster 1 (“Metabolically Healthy Young Adults”) closely resembles EOSS stage 0–1, where patients have obesity without overt complications or only mild risk factors. This reinforces the importance of early intervention aimed at preventing disease progression, consistent with ABCD principles that prioritize early intervention to alter long-term trajectories rather than waiting for complications to emerge.

At the other end of the spectrum, Cluster 4 (multimorbid senior adults”) and Cluster 5 (older—advanced age, highly comorbid individuals) align with EOSS stages 3–4, characterized by established end-organ damage, functional limitations, and high cardiovascular risk. These clusters illustrate the advanced stages of ABCD, where adiposity-related complications dominate clinical management and require intensive, multimodal care. Importantly, the coexistence of social disadvantage in Cluster 4 highlights a dimension not explicitly captured in EOSS staging but highly relevant to outcomes, suggesting that future refinements of staging systems may benefit from integrating social determinants of health.

The observed mismatch between disease burden and treatment utilization in some clusters further underscores the clinical utility of EOSS and ABCD frameworks. For instance, the relatively low use of nutritional counselling in highly comorbid older adults (Cluster 5) appears discordant with their likely high EOSS stage and associated need for comprehensive care. Conversely, the limited use of bariatric surgery in earlier-stage patients (e.g., Cluster 1) raises questions about whether current practice sufficiently leverages the potential benefits of early, complication-preventing interventions, as increasingly advocated within the ABCD model.

Taken together, our clustering approach complements established staging systems by offering an empirical, population-level perspective on obesity heterogeneity while supporting the central premise of both ABCD and EOSS: that obesity management should be individualized, complication-driven, and aligned with disease severity rather than BMI alone.

### 4.6. Future Directions

Future longitudinal studies are needed to determine whether the phenotypes that have emerged in our study are associated with differences in treatment response, treatment persistence, healthcare utilization, and clinical outcomes, and whether cluster-based classification provides prognostic or predictive information beyond established approaches such as BMI and EOSS. Further studies should also evaluate whether cluster-informed approaches can improve clinical decision-making, including the timing and selection of lifestyle interventions, pharmacotherapy, bariatric surgery referral, and multidisciplinary care pathways. Integration of additional domains, such as dietary patterns, psychological factors, sleep characteristics, environmental exposures, and detailed measures of healthcare access, may further enhance the ability of clustering approaches to capture the complexity of obesity, and advance personalized treatment strategies in return.

### 4.7. Limitations

This study has several limitations. First, our study relied on administrative registry data, which may be subject to coding errors or incomplete documentation. Several variables are self-reported, which are subject to recall bias and to social desirability bias, such as conduct of physical activity, smoking and group therapy attendance. Second, although we identified distinct clusters, the cross-sectional design limits casual interpretation and prevents assessment of longitudinal weight trajectories, treatment response, or the effectiveness of specific interventions within these clusters. Therefore, associations between cluster memberships, treatment patterns and clinical characteristics should be considered hypothesis-generating and require validation in prospective studies. Third, the study was conducted within a single Israeli healthcare system. Differences in population demographics, socioeconomic structure, healthcare organization, prescribing practices, and access to obesity care may limit the generalizability of the identified phenotypes to other settings. External validation in independent populations and healthcare systems is therefore required. Fourth, several variables were not available in our dataset. Among those variables are potential determinants of obesity heterogeneity, including dietary patterns, sleep characteristics, and psychological factors, that may influence obesity phenotypes and clinical outcomes. In addition, indications for dietitian encounters and medication exposure and dosage information (particularly for GLP-1 receptor agonists), were not available. Consequently, these interventions could not be reliably attributed specifically to obesity treatment and may have reflected management of diabetes or other approved indications. Finally, BMI was used as the primary inclusion criterion, which may misclassify adiposity and does not fully capture disease severity, organ dysfunction, or metabolic risk [3,4].

## 5. Conclusions

The identification of five distinct multidimensional obesity phenotypes in the Israeli population underscores the necessity of moving beyond BMI as the sole guide for treatment. Our results highlight two divergent clinical priorities: early, preventive engagement for the “Metabolically Healthy Young Adults” cluster to maximize their naturally high potential for treatment success, and intensive, socially supported management for the “Sick and Low SES” cluster, where medical complexity and social disadvantage may highly benefit from integrated care approaches. Recognizing these profiles allows for a more efficient allocation of healthcare resources and support individualized treatment strategies. Ultimately, clustering approaches should be considered complementary to established obesity frameworks by providing an additional lens through which clinicians and researchers can understand patient heterogeneity. Future validation studies will determine whether these data-driven phenotypes improve risk prediction, guide therapeutic choices, and contribute meaningfully to precision obesity medicine.

## Figures and Tables

**Table 1:** Baseline demographic and clinical characteristics of the study population by obesity cluster.

Cluster	n	Mean Age (SD)	Sex— Male %	Mean Charlson Index	General Jewish %	Smoke %	PA (Yes) %	Low SES %	High SES %	Mean BMI
(1) Metabolically healthy Young Adults	12,997	26.4 (4.38)	35	0.07	36	18	23	46	1.5	34.94
(2) Adults with Low Comorbidity	12,159	40 (3.75)	38	0.72	44	20	23	40	3	34.9
(3) Middle-Aged with Moderate Comorbidity	15,793	53.2 (3.65)	42	2.44	51	20	22	37	3	34.8
(4) Multimorbid Seniors with Socioeconomic Disadvantage	16,669	65.5 (3.36)	42	4.29	66	15	22	44	2	34.31
(5) High Clinical Burden Advanced-Age Adults with higher SES	10,585	77.2 (4.95)	39	6.49	78	8	21	20	7	34.05

Abbreviations: SD, Standard Deviation; PA, Physical Activity; SES, Socioeconomic Status; BMI, Body Mass Index; Positive PA = at least 1 h/week (categories 3 + 4). SES—defined by AGAS. High SES = AGAS 15–20.

**Table 2:** Mean values of clinical measures and proportions of clinical comorbidities by cluster.

Cluster	HTN	COPD	CVA	DM	DM with End Organ Damage	Glucose (Mean, mg/dL)	HbA1C (Mean, %)	TG (Mean, mg/dL)	Dyslipidemia	ADHD	IHD	OSA
(1) Metabolically healthy Young Adults	3	0	0	3	0	92.55	5.15	116.56	6	17	0	2
(2) Adults with Low Comorbidity	12	2	0	13	2	99.8	5.46	144.59	22	7	1	4
(3) Middle-Aged with Moderate Comorbidity	38	5	2	31	7	110.24	5.93	153.45	50	3	6	8
(4) Multimorbid Seniors with Socioeconomic Disadvantage	66	11	5	43	13	113.75	6.09	142.39	72	2	13	12
(5) High Clinical Burden Advanced Age Adults with higher SES	85	16	8	56	22	115.53	6.17	131.75	81	0	23	13

Abbreviations: HTN, Hypertension; COPD, Chronic Obstructive Pulmonary Disease; CVA, Cerebrovascular Accident; DM, Diabetes Mellitus; HbA1C, Hemoglobin A1C; TG, Triglycerides; ADHD, Attention-Deficit/Hyperactivity Disorder; IHD, Ischemic Heart Disease; OSA, Obstructive Sleep Apnea.

**Table 3:** Treatment modality by obesity clusters, number of individuals.

Treatment Modality	(1) Metabolically Healthy Young Adults (n = 12,997; 19%)	(2) Adults with Low Comorbidity (n = 12,159; 18%)	(3) Middle-Aged with Moderate Comorbidity (n = 15,793; 23%)	(4) Multimorbid Seniors with Socioeconomic Disadvantage (n = 16,669; 24%)	(5) High Clinical Burden Advanced Age Adults with Higher SES (n = 10,585; 16%)
Drug—GLP-1, n = 10,888	1460	716	3140	3551	2021
Drug—Phentermine, n = 4	0	3	1	0	0
Drug—Orlistat, n = 1	0	0	1	0	0
Bariatric surgery, n = 340	148	91	78	21	2
Dietitian consultation/follow-up, n = 11,089	2439	2544	2840	2157	1109
Weight-loss workshop, n = 2300	589	622	563	372	154

## Data Availability

The data supporting the findings of this study are not publicly available because they contain information subject to ethical and privacy restrictions. De-identified data may be made available by the corresponding author upon reasonable request, subject to approval by the relevant ethics committee and the applicable data-sharing agreements.

## Supplementary Materials

The following supporting information can be downloaded at: https://www.mdpi.com/article/10.3390/jcm15187026/s1, Table S1: STROBE checklist for the cross-sectional study; Table S2: Additional cluster validation indices, based on numeric variables; Figure S1: R-Squared by number of clusters, based on numeric variables.
