# Supplementary material for: Population-Level Heterogeneity in People with Obesity: A Cross-Sectional Cluster Analysis of a Population-Based Registry
Source: J Clin Med. 2026 Sep 10;15(18):7026. doi: 10.3390/jcm15187026 (PMC13607413; doi:10.3390/jcm15187026)

Supplementary Table S1. STROBE checklist for the cross-sectional study

Section/topic	Item	STROBE reporting requirement	Location in manuscript	Assessment / action
Title and abstract	1	Identify the observational study design and provide a balanced summary of methods and findings.	Title; Abstract	Reported.
Introduction: Background/rationale	2	Explain the scientific background and rationale.	Introduction, paragraphs 1–2	Reported.
Introduction: Objectives	3	State the specific study objectives and any prespecified hypotheses.	Introduction, final paragraph	Reported. No prespecified hypothesis is stated, consistent with the exploratory clustering design.
Methods: Study design	4	Present the key elements of the study design early.	Methods, “Study design, setting, and population”	Reported. The study is explicitly described as cross-sectional.
Methods: Setting	5	Describe the setting, location, and relevant dates for data collection and study conduct.	Methods, “Study design, setting, and population”	Reported.
Methods: Participants	6	Describe eligibility criteria and the source and method of participant selection.	Methods, “Study design, setting, and population”; Results, “Study Population”	Reported.
Methods: Variables	7	Clearly define variables of interest and relevant diagnostic criteria.	Methods, “Variables”; Tables 1–3	Largely reported. Demographic, socioeconomic, lifestyle, clinical, laboratory, and treatment-related variables are described. Comorbidities are derived from documented diagnoses in the electronic record using ICD-9 coding.
Methods: Data sources and measurement	8	Describe the source and assessment of each variable and whether assessment methods were comparable across groups.	Methods, “Study design, setting, and population” and “Variables”	Reported.
Methods: Bias	9	Describe efforts to address potential sources of bias.	Methods, “Analysis”; Discussion, “Limitations”	Missing categorical data were retained as a separate category, and limitations related to coding, incomplete documentation, recall bias, and social desirability bias are acknowledged.
Methods: Study size	10	Explain how the study size was determined.	Methods, “Study design, setting, and population”	Reported.
Methods: Quantitative variables	11	Explain how quantitative variables were handled, including any categorization.	Methods, “Variables” and “Analysis”; Table 1 footnotes	Reported.
Methods: Statistical methods	12	Describe statistical methods, missing-data handling, subgroup methods, and sensitivity analyses where applicable.	Methods, “Analysis”	Reported.

Section/topic	Item	STROBE reporting requirement	Location in manuscript	Assessment / action
Results: Participants	13	Report the number of individuals at each stage and reasons for exclusion/nonparticipation; consider a flow diagram.	Methods, “Study design, setting, and population”; Results, “Study Population”	Partially reported. The registry contained 114,028 individuals and 68,203 were analyzed. We explicitly state the reason the remaining registry members were not included.
Results: Descriptive data	14	Report participant characteristics and the amount of missing data for variables of interest.	Results, “Study Population” and “Characteristics of patient profiles”; Tables 1–2	Participant characteristics are reported in detail for the cohort and clusters.
Results: Outcome data	15	For a cross-sectional study, report relevant summary measures.	Results, “Identification of obesity clusters,” “Characteristics of patient profiles,” and “Treatment utilization patterns”; Tables 1–3	Reported.
Results: Main results	16	Report the principal estimates and category boundaries where relevant; provide adjusted estimates if applicable.	Results; Tables 1–3	Reported for this descriptive clustering study.
Results: Other analyses	17	Report subgroup, interaction, or sensitivity analyses that were performed.	Results	Reported.
Discussion: Key results	18	Summarize the main findings in relation to the study objectives.	Discussion, opening paragraph	Reported.
Discussion: Limitations	19	Discuss limitations and potential sources of bias or imprecision.	Discussion, “Limitations”	Reported.
Discussion: Interpretation	20	Provide a cautious interpretation in light of objectives, limitations, multiplicity, and prior evidence.	Discussion, throughout	Generally reported, with comparison to prior clustering studies and ABCD/EOSS frameworks.
Discussion: Generalizability	21	Discuss external validity and generalizability.	Discussion, “Limitations”	Reported.
Other information: Funding	22	State the source of funding and the role of the funder, where applicable.	End matter, “Financial support”	Funding source and grant number are reported.

Table S2: Additional cluster validation indices, based on numeric variables

Number of clusters	Cost	R_squared	Cubic Clustering Criterion
3	4,615,180	0.794	−0.895
4	3,695,063	0.837	−0.969
**5**	3,152,489	0.862	−1.035
6	2,876,803	0.875	−1.131
7	2,661,773	0.885	−1.208
8	2,483,180	0.893	−1.271
9	2,317,588	0.901	−1.316
10	2,172,397	0.907	−1.352

Figure S1: R-Squared by number of clusters, based on numeric variables

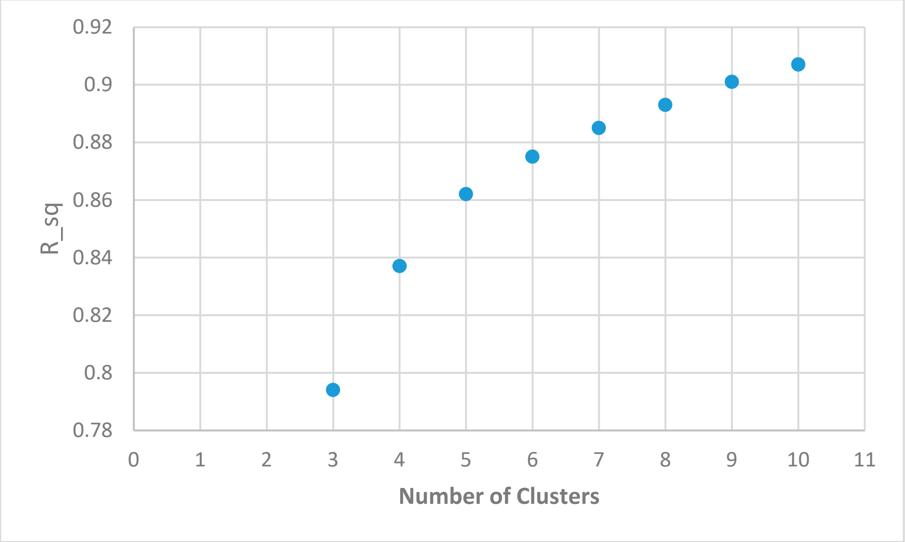

Supplement: Supplementary file 1 [file jcm-15-07026-s001.zip › jcm-4465047-supplementary.pdf]
